# THE EXPERIENCES OF OLDER ADULTS IN HAITI AND THE UNITED STATES AT THE ONSET OF COVID-19

**DOI:** 10.1093/geroni/igad104.3640

**Published:** 2023-12-21

**Authors:** Laurie Blackman, Kathryn Krase, Donna Wang

**Affiliations:** Yeshiva University, New York City, New York, United States; Krase Consulting, Brooklyn, New York, United States; Springfield College, Springfield, Massachusetts, United States

## Abstract

From the early onset of the COVID-19 pandemic, there was a disproportionate risk and impact of the disease on older adults and communities of color. This article explores the differences in experiences of older Haitian and older US adults at the onset of the COVID-19 pandemic. Since wealth creates accessibility to needed resources, including the best healthcare and support services, the disproportionate risk and impact of COVID-19 on older adults required a focused look at how older adults in vulnerable countries such as Haiti faired when compared to older adults in the United States. Using data collected from a sample of 240 Haitian older adults in August 2020 and 204 US older adults in June 2020, the findings indicated that Haitian older adults experienced more significant psychological, financial, and emotional distress due to the COVID-19 outbreak than their US older adult counterparts. The results of this study revealed the challenges that older adults faced at the onset of the pandemic, which was more pronounced in developing countries. However, the surge in violence in Haiti in the first quarter of 2023 killed more people than the COVID-19 outbreak. This research provides important insight for developing and framing policies and interventions that are not modeled on the measures taken elsewhere but adapted to a country’s reality and the population’s actual needs.

